# Supplementary material for: Enhanced Epithelial-to-Mesenchymal Transition and Chemoresistance in Advanced Retinoblastoma Tumors Is Driven by miR-181a
Source: Cancers (Basel). 2022 Oct 19;14(20):5124. doi: 10.3390/cancers14205124 (PMC9600213; doi:10.3390/cancers14205124)
Supplement: Supplementary file 1 [file cancers-14-05124-s001.zip › cancers-1949861-supplementary.pdf]

Supplementary data

Figure S1

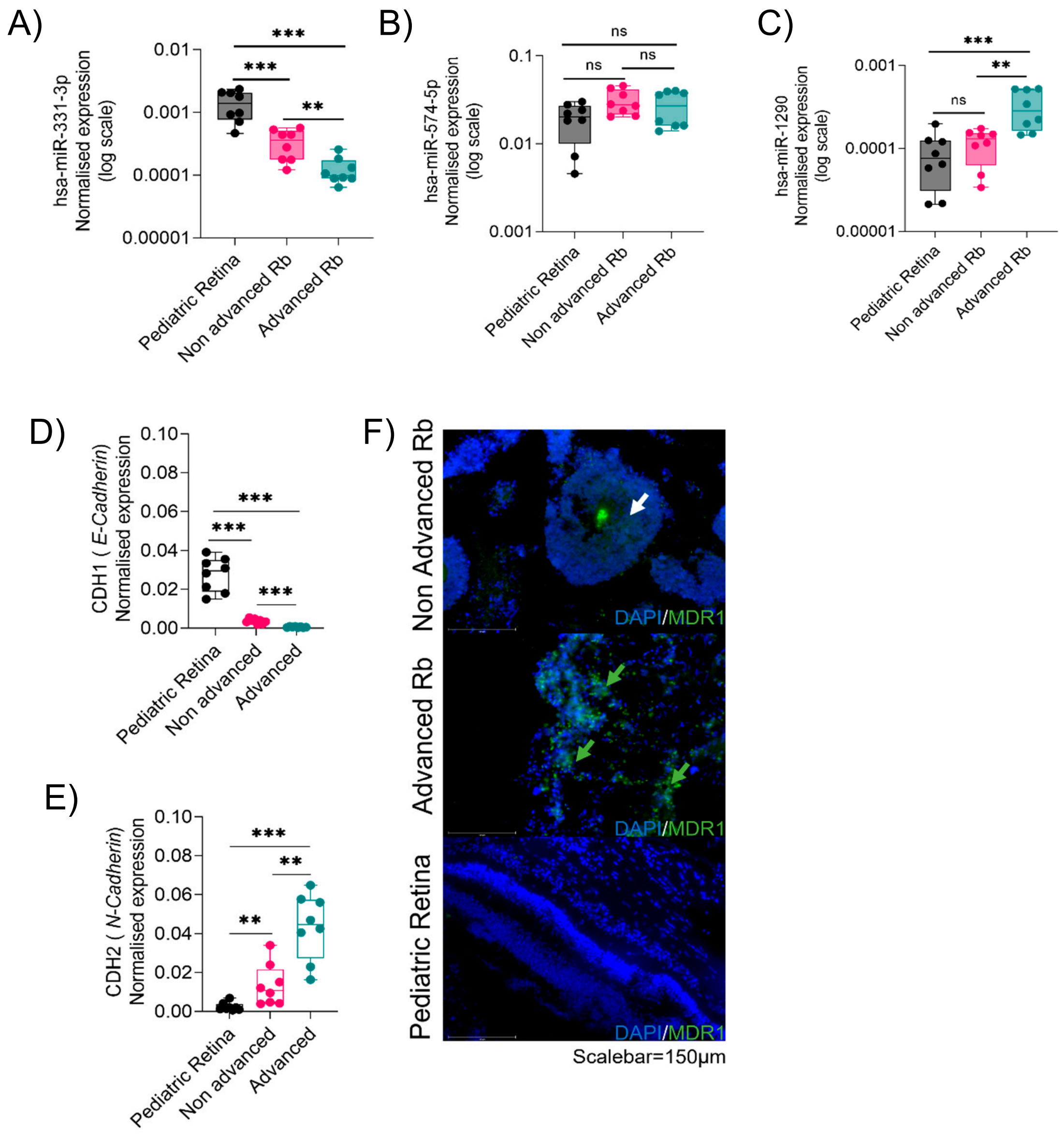

Figure S1. Transcriptomic profiling identifies differentially regulated miRNAs, EMT, and drug-resistant genes in Rb tumor subtypes. RT-PCR validations of microarray identified miRNAs (A) has-miR-331-3p (B) has-miR-574-5p (C) has-miR-1290 in advanced (n=4), non-advanced (n=4) and control pediatric retina (n=4). RT-PCR validations for microarray identified mRNAs (D) CDH1 and (E) CDH2 in advanced (n=4), non-advanced (n=4) and control pediatric retina (n=4) (F) Immunofluorescence showing MDR1 expression in advanced Rb (n=4), non-advanced Rb (n=4) and pediatric retina tissues (n=4). Scale bar =150µm. Values represent mean ± s.d. Two-tailed Mann-Whitney was used for statistical analysis., $**P < 0.01$, $***P < 0.001$. 'ns' represents no statistically significant difference between the means of two variables.

Figure S2:

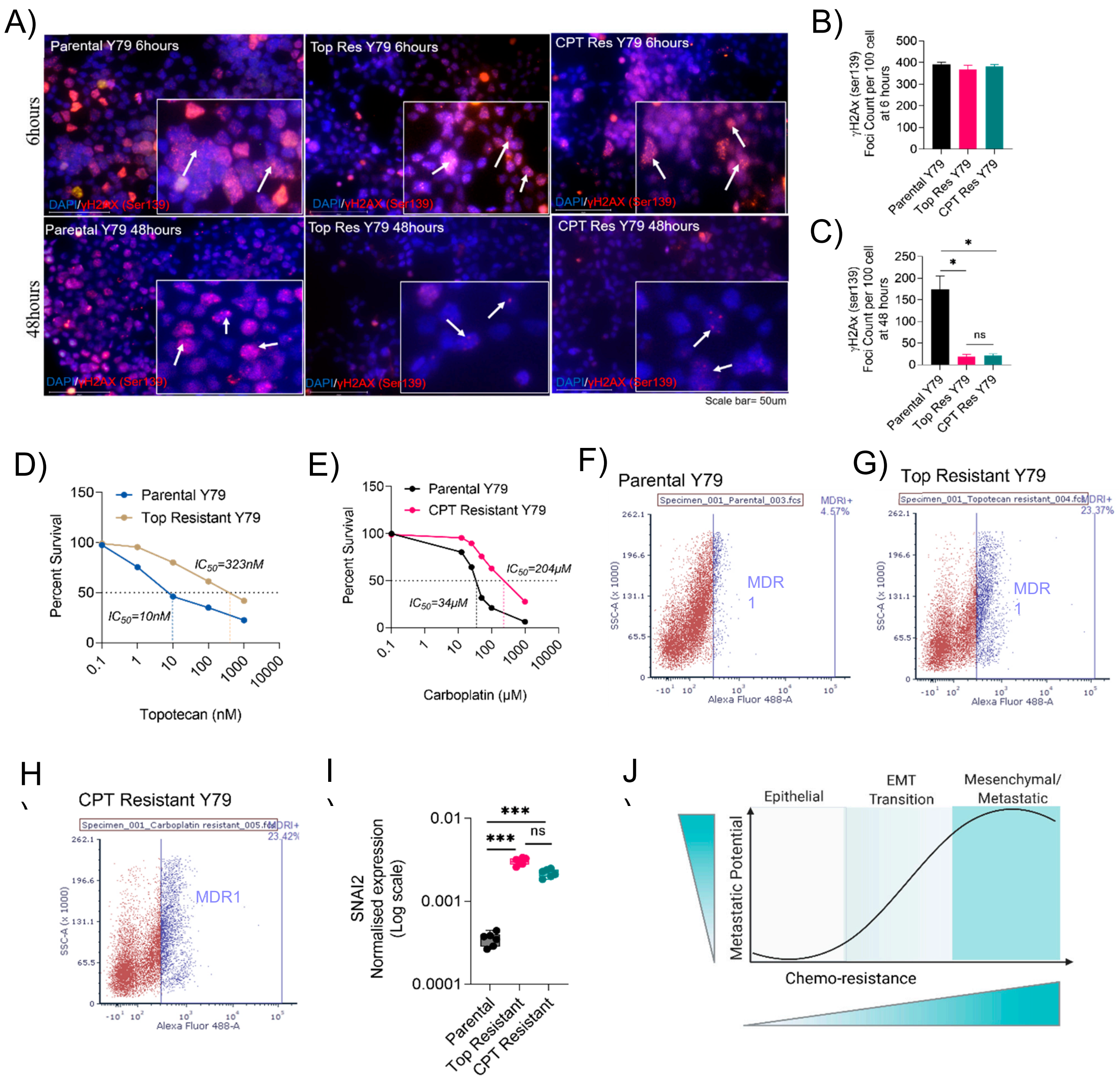

Figure S2. Chemotherapy-resistant Rb cells confer high EMT program and metastasis**.** (A) Immunofluorescence showing λH2A.x foci in parental, topotecan resistant and carboplatin resistant cells upon IC50 dose treatment using topotecan or carboplatin for 6 hours to 48hours. λH2A.x foci count at (B) 6 hours and (C) 48hours of topotecan and carboplatin therapy. Survival assay to determine the IC50 shift in resistant lines with increasing concentration of (D) topotecan (E) carboplatin. MDR1 surface staining analyzed by flow cytometry in (F) Parental Y79 cells (G) Topotecan resistant Y79 cells (H) Carboplatin

resistant Y79 cells. (I) RT-PCT results showing expression of SNAI2 in parental, topotecan resistant and carboplatin resistant cells. (J) Schematic showing EMT trans-differentiation and induction of drug resistance transit the cells to a dedifferentiated mesenchymal/ drug-resistant metastatic phenotype. Values represent mean ± s.d. Two-tailed Mann-Whitney was used for statistical analysis. *$P < 0.05$, , ***$P < 0.001$. 'ns' represents no statistically significant difference between the means of two variables.

Figure S3:

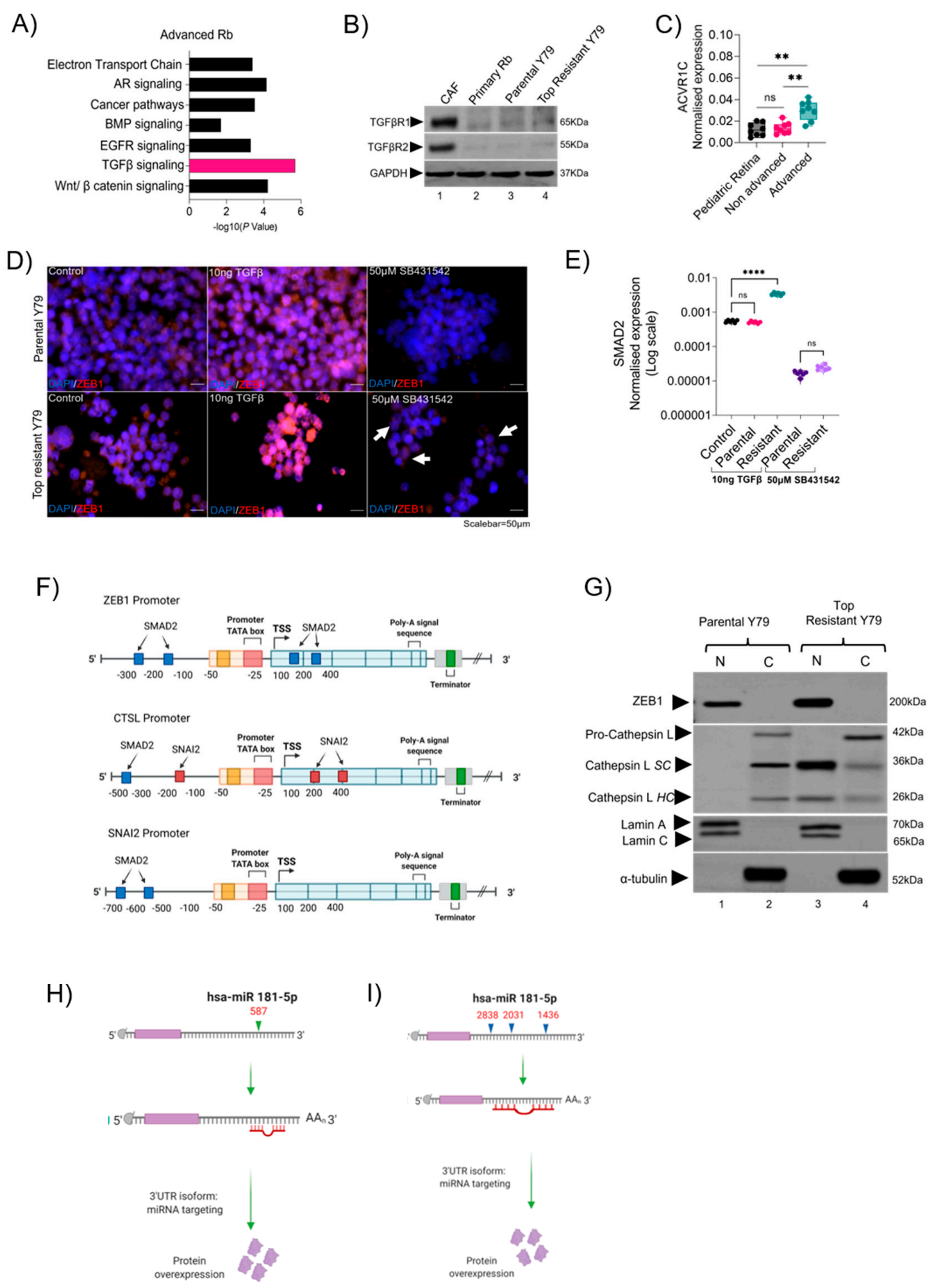

Figure S3. Resistant cells elicit transition through ZEB1 and resistance through Cathepsin L. (A) KEGG pathway enrichment analysis showing differentially regulated pathways in advanced Rb tumors. (B) Immunoblot showing the expression of canonical TGFβ receptors I and II in retinoblastoma associated fibroblast (CAF) primary culture, T4a stage Rb tumor primary culture,

parental Y79 and topotecan resistant Y79. (C) RT-PCR results showing normalized expression of ACVR1C receptors in pediatric retina (n=4), advanced (n=4) and non-advanced Rb tumors(n=4). (D) Immunofluorescence showing ZEB1 expression upon TGFβ induction and TGFβ inhibition in parental and topotecan resistant Y79 cells for 48hours. Scalebar=50μm. (E) RT-PCR showing normalized expression of SMAD2 upon TGFβ induction and TGFβ inhibition in parental and topotecan resistant Y79 cells for 48hours. (F) Schematic showing promoter binding regions of SMAD2 in ZEB1 promoter, SMAD2 and SNAI2 in CTSL promoter and SMAD2 in SNAI2 promoter. The binding sites in each promoter are curated using euakaryotic promoter database. (G) Nuclear-cytoplasmic fraction immunoblot showing the subcellular localization of ZEB1 and CTSL in parental and resistant Y79 cells. MicroRNA target prediction database (miRwalk and Targetscan) predicted binding regions of miR-181a-5p in (H) ZEB1 3'UTR (I) SNAI2 3'UTR. Values represent mean ± S.E.M. Two-tailed Student's t-test (for 2 groups) and one-way ANOVA with Dunnett's multiple comparisons tests (for >2 group) were used for statistical analysis. $^{**}P < 0.01$,. $^{****}P < 0.0001$. 'ns' represents no statistically significant difference between the means of two variables

Figure S4:

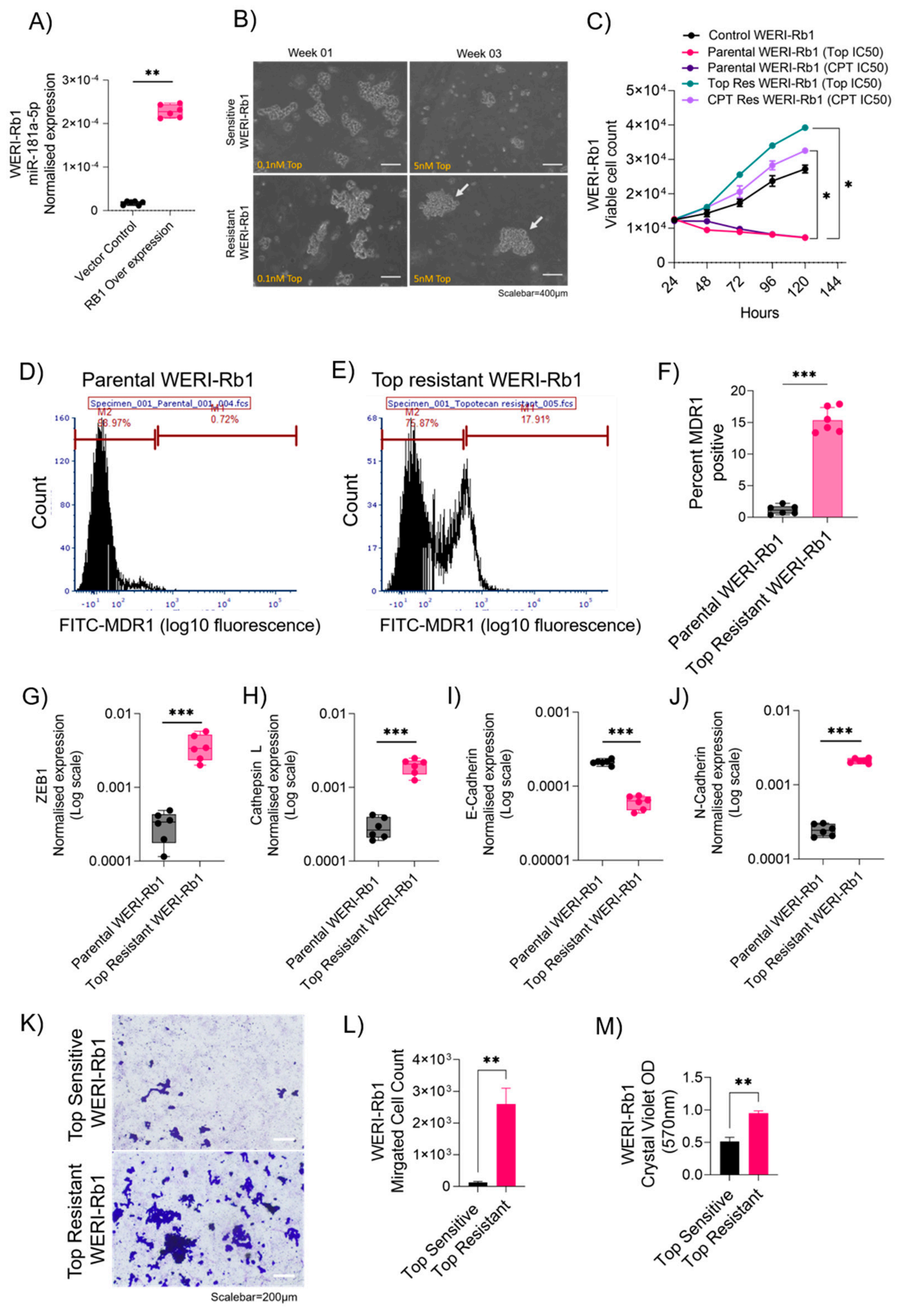

A)
WERI-Rb1 miR-181a-5p Normalised expression
Vector Control
RB1 Over expression
B)
Week 01
Week 03
Sensitive WERI-Rb1
Resistant WERI-Rb1
0.1nM Top
5nM Top
Scalebar=400µm
C)
Control WERI-Rb1
Parental WERI-Rb1 (Top IC50)
Parental WERI-Rb1 (CPT IC50)
Top Res WERI-Rb1 (Top IC50)
CPT Res WERI-Rb1 (CPT IC50)
WERI-Rb1 Viable cell count
Hours
D)
Parental WERI-Rb1
Count
FITC-MDR1 (log10 fluorescence)
E)
Top resistant WERI-Rb1
Count
FITC-MDR1 (log10 fluorescence)
F)
Percent MDR1 positive
Parental WERI-Rb1
Top Resistant WERI-Rb1
G)
ZEB1 Normalised expression (Log scale)
H)
Cathepsin L Normalised expression (Log scale)
I)
E-Cadherin Normalised expression (Log scale)
J)
N-Cadherin Normalised expression (Log scale)
K)
Top Sensitive WERI-Rb1
Top Resistant WERI-Rb1
Scalebar=200µm
L)
WERI-Rb1 Mirgated Cell Count
Top Sensitive
Top Resistant
M)
WERI-Rb1 Crystal Violet OD (570nm)
Top Sensitive
Top Resistant

Figure S4. Chemotherapy-resistant WERI-Rb1ells confer high EMT program and metastasis. (A) RT-PCR showing normalized expression of miR-181a-5p in Vector control (RB1 null) and RB1 over expressed WERI-Rb1 retinoblastoma cells. (B) Phase contrast microscopy images showing morphology of parental and resistant WERI-Rb1 cells under increasing dose of topotecan treatments from week1 to week3. Scalebar=400µm. (C) Trypan blue cell count of parental, topotecan resistant and carboplatin resistant WERI-Rb1 cells for 24hr, 48hr, 72hr and 96hr. MDR1 surface expression analysis in (D) parental and (E) topotecan resistant WERI-Rb1 cells by flow cytometry. (F) Bar graph showing the percentage of cells positive for MDR1 surface expression in parental and topotecan resistant WERI-Rb1 cells. RT-PCR showing expression of (G) ZEB1 (H) Cathepsin L (I) E-cadherin and (J) N-cadherin in parental and topotecan resistant WERI-Rb1 cells. (K) Transwell invasion and migration assay to assess the migratory capacity of resistant cells compared to sensitive cells under 10nM topotecan treatment for 48hours. (L) Crystal violet OD reading at 570nm to assess invasiveness (M) Trypan blue count to assess migrated cells in the lower compartment of the transwell chamber. Values represent mean ± S.D. Two-tailed Student's t-test (for 2 groups) and one-way ANOVA with Dunnett's multiple comparisons tests (for >2 group) were used for statistical analysis. $*P < 0.05$, $**P < 0.01$, $***P < 0.001$.

Figure S5:

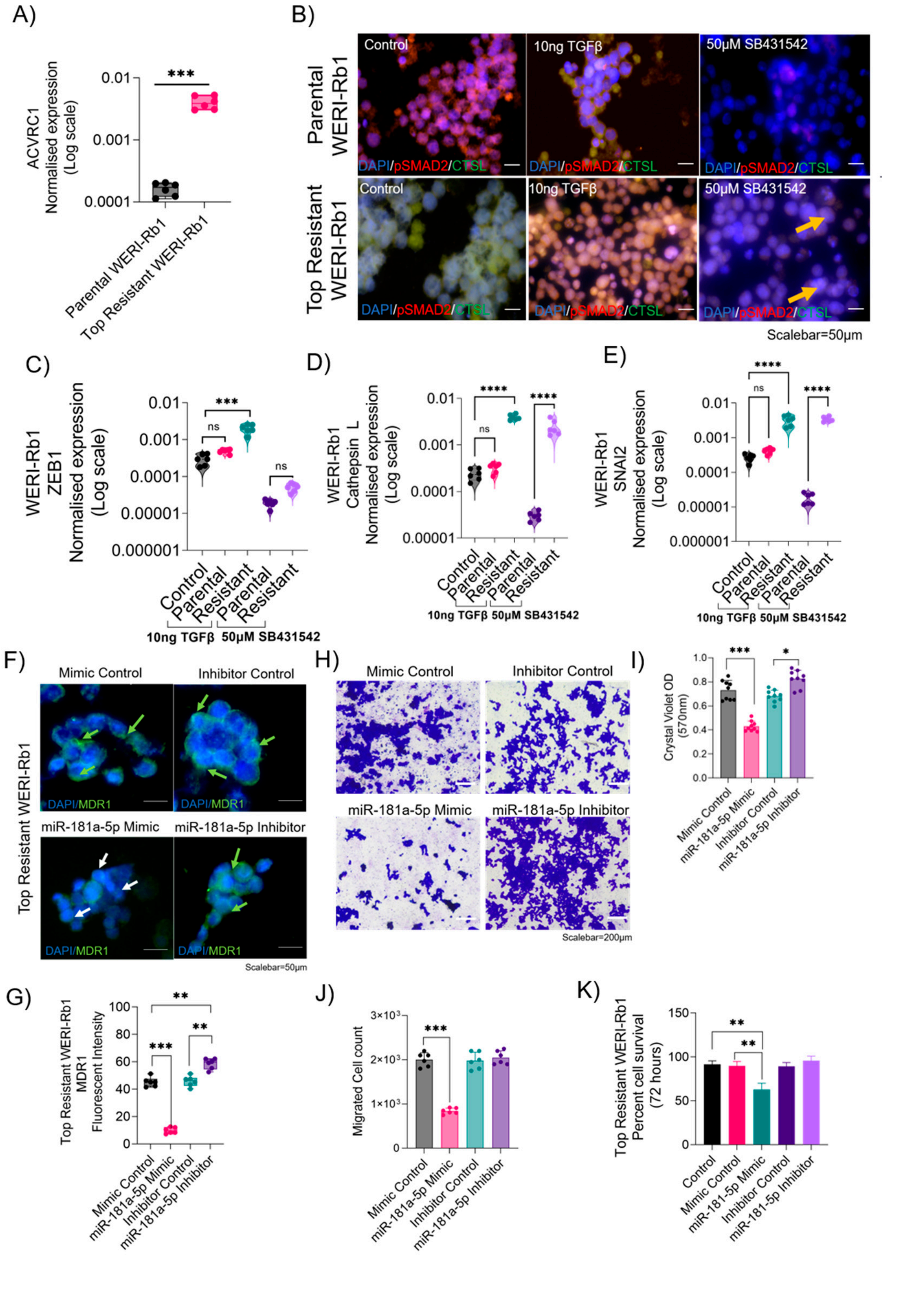

Figure S5. Resistant WERI-Rb1 cells elicit transition through ZEB1 and resistance through Cathepsin L and resistance depletion by miR-181a-5p confers sensitivity to chemotherapy (A) RT-

PCR results showing expression of ACVRC1 in parental, topotecan resistant and carboplatin resistant cells. (B) Immunofluorescence showing expression of phospho-SMAD2 and cathepsin L (CTSL) upon TGFβ induction (10ng for 48 hours) and TGFβ inhibition (50μM SB431542 for 48 hours) in parental and topotecan resistant WERI-Rb1 cells. Scalebar=50μm. RT-PCR results show normalized expression of (C) ZEB1 (D) CTSL (E) SNAI2 upon TGFβ induction and inhibition for 48hours. (F) Immunofluorescence showing MDR1 surface expression in topotecan resistant WERI-Rb1 cells upon miR-181a-5p overexpression and inhibition. Scalebar=50μm. (G) Bar graphs showing MDR1 fluorescent intensity in topotecan resistant WERI-Rb1 cells upon miR-181a-5p overexpression and inhibition. (H)Transwell invasion and migration assay to assess the invasive and migratory capacity of topotecan resistant cells upon miR-181a-5p overexpression and inhibition (I) Crystal violet OD measurement at 570nm to assess the invasiveness of resistant WERI-Rb1 cells. (J) Trypan blue cell count shows migrated cells in the lower compartment of the transwell chamber. (K) Chemosensitivity of miR-181a-5p modulated topotecan resistant WERI-Rb1 cells upon 10nM topotecan treatment for 72 hours. Values represent mean ± S.D. Two-tailed Student's t-test (for 2 groups) and one-way ANOVA with Dunnett's multiple comparisons tests (for >2 groups) were used for statistical analysis. *$P < 0.05$, **$P < 0.01$, ***$P < 0.001$, ****$P < 0.0001$. 'ns' represents no statistically significant difference between the means of two variables.

Figure S6: Statistical decision tree

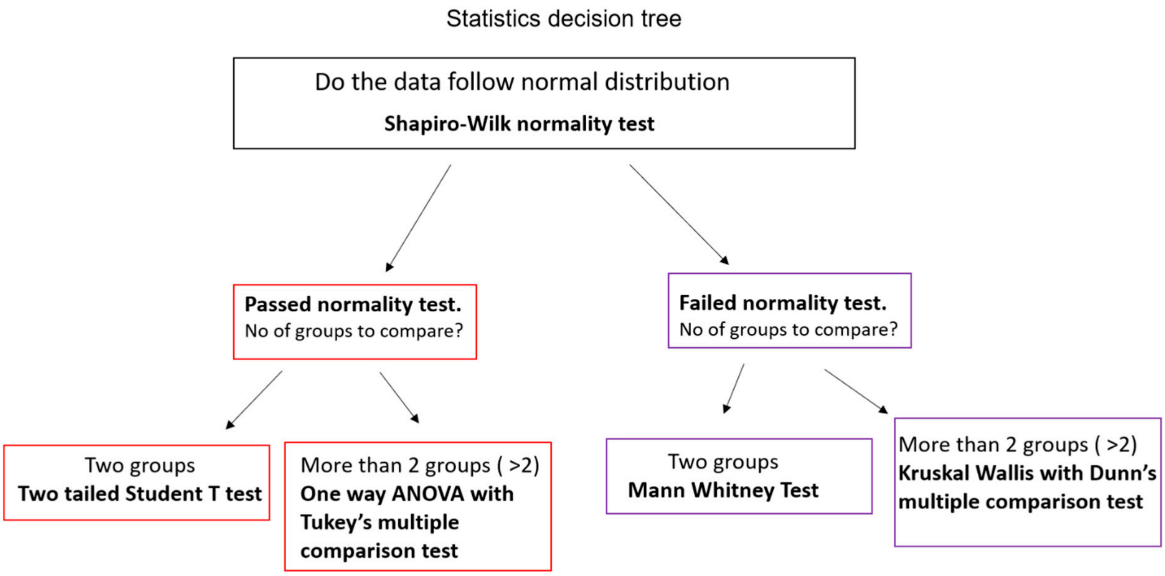

Figure S7: Uncropped blots of Figure 5A

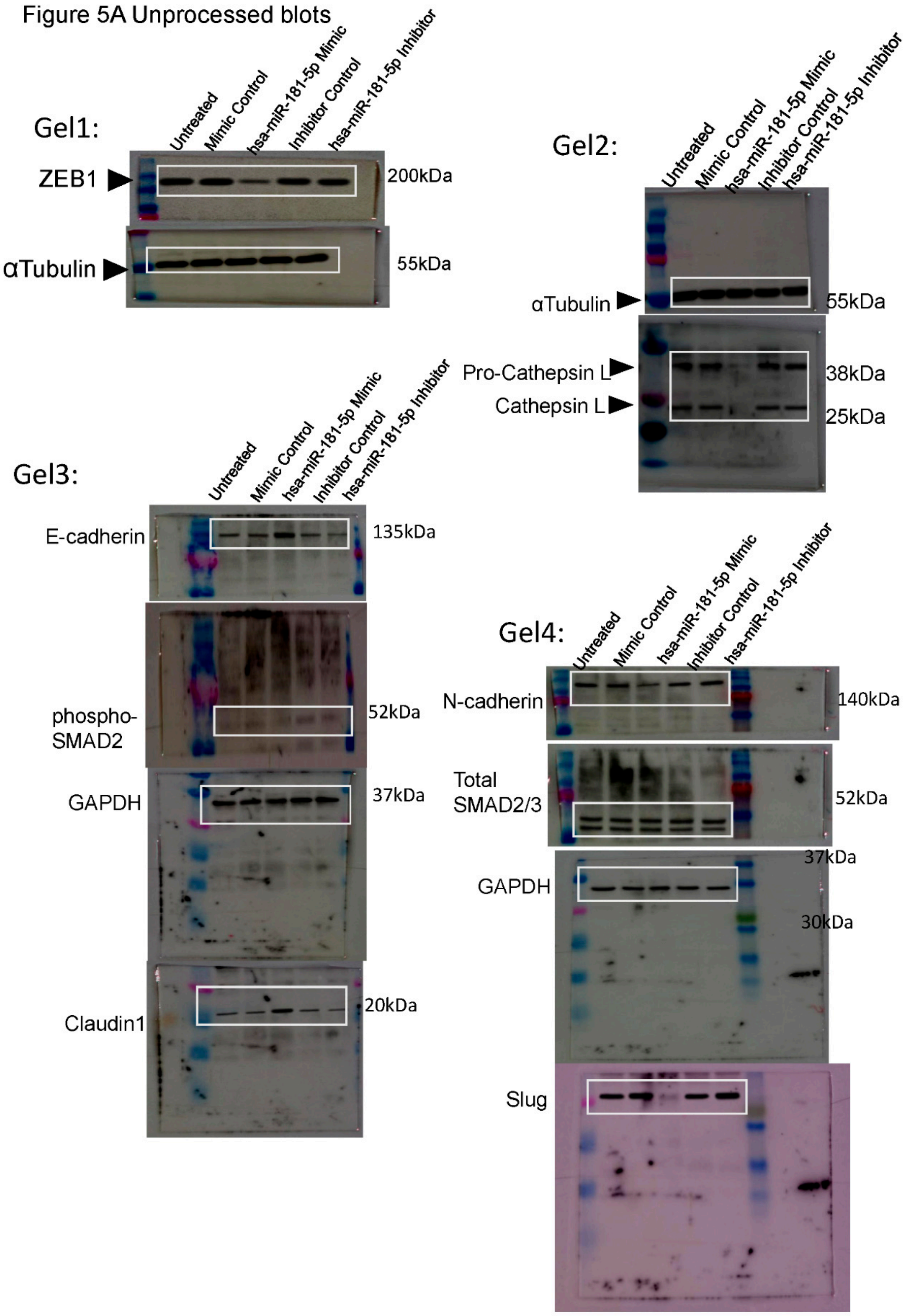

Figure S8: Uncropped immunoblots of Figure 4A

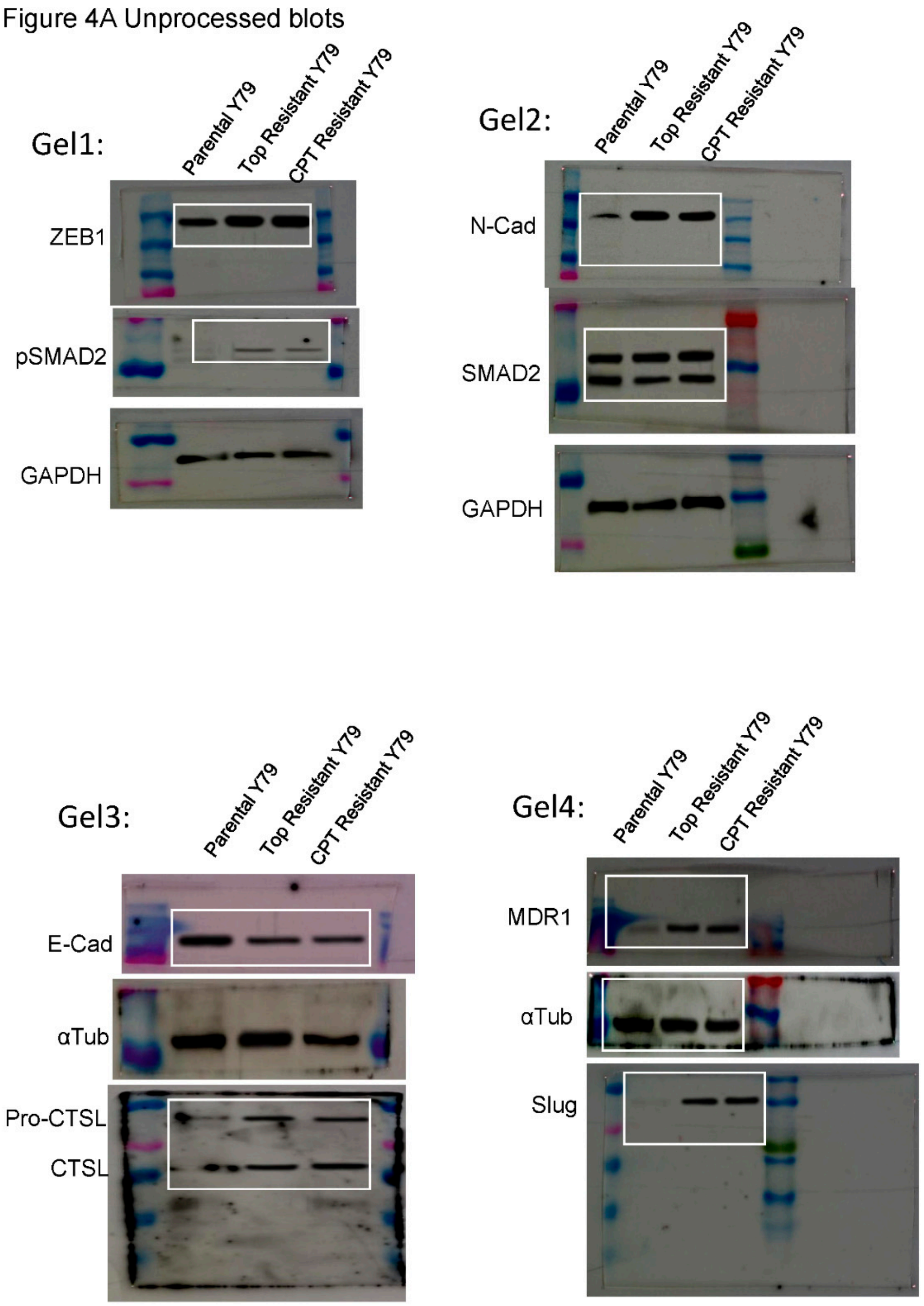

Figure S9: Uncropped immunoblots of Figure 4E

Figure 4E Unprocessed blots

Gel1:

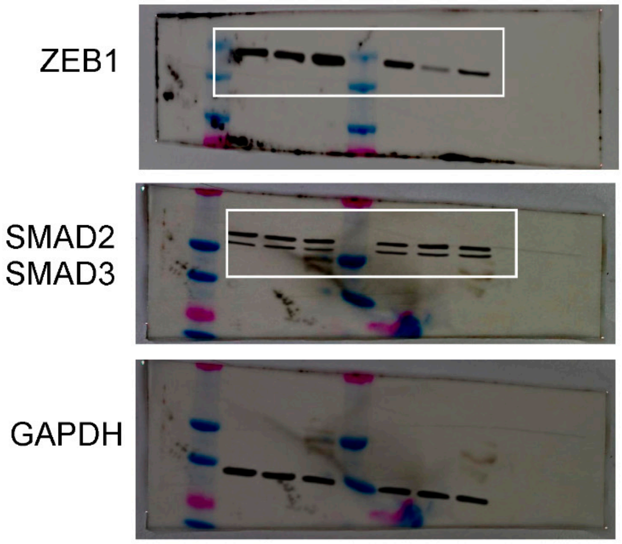

Gel1:

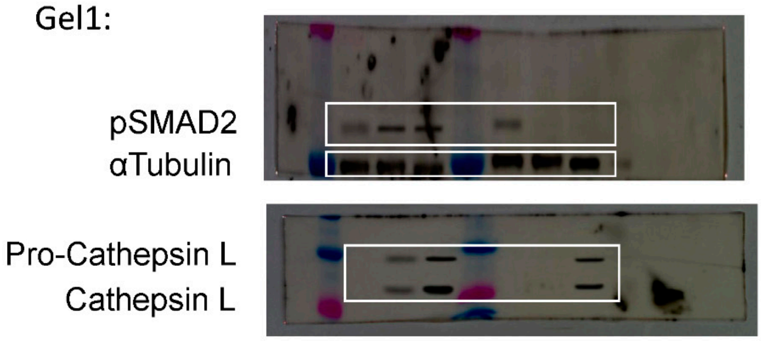

Table S1: Clinical and histopathological details of samples used for RT-PCR validations

ID	Sex	Age at presentation	Laterality	Clinical Risk	IIRC Group	AJCC staging
P10	F	23 months	Bilateral	Advanced	Group E	cT3b
P11	F	24month	Unilateral	Advanced	Group E	cT3b
P12	M	36 months	Bilateral	Advanced	Group E	cT3b
P13	F	33 months	Unilateral	Advanced	Group E	cT3a
P14	M	7 months	Bilateral	Non-advanced	Group D	cT2a
P15	M	30 months	Bilateral	Non-advanced	Group D	cT2b
P16	F	14months	Unilateral	Non-advanced	GroupD	cT2b
P17	M	11 months	Unilateral	Non-advanced	Group D	cT2b
C1	M	2 months		Multiple organ dysfunction (No ocular complications)		
C2	F	12 months		No ocular complications		
C3	M	3 months		No ocular complications		
C4	M	6 months		No ocular complications		

Table S2: Clinical and histopathological details of additional Rb samples used for immunohistochemistry validations

ID	Sex	Age at presentation	Laterality	Clinical Risk	IIRC Group	AJCC staging
P10	F	23 months	Bilateral	Advanced	Group E	cT3b
P11	F	24month	Unilateral	Advanced	Group E	cT3b
P12	M	36 months	Bilateral	Advanced	Group E	cT3b
P13	F	33 months	Unilateral	Advanced	Group E	cT3a
P14	M	36 months	Unilateral	Advanced	Group E	cT3b
P15	M	48 months	Unilateral	Advanced	Group E	cT3b
P16	F	33 months	Unilateral	Non-advanced	Group D	cT2b
P17	F	14 months	Bilateral	Non-advanced	Group D	cT2b
P18	M	11 months	Unilateral	Advanced	Group E	cT3b
P19	M	3 months	Unilateral	Advanced	Group E	cT3b
P20	M	33 months	Unilateral	Advanced	Group E	cT3a
P21	F	45months	Bilateral	Advanced	Group E	cT3b
P22	M	7 months	Bilateral	Non-advanced	Group D	cT2a
P23	M	30 months	Bilateral	Non-advanced	Group D	cT2b
P24	F	14months	Unilateral	Non-advanced	GroupD	cT2b
P25	M	11 months	Unilateral	Non-advanced	Group D	cT2b
P26	M	25 months	Bilateral	Advanced	Group E	cT3b
P27	F	20 months	Bilateral	Advanced	Group E	cT3b
P28	F	24 months	Bilateral	Non-advanced	Group D	cT2a
P29	F	36 months	Bilateral	Non-advanced	Group D	cT2b
P30	M	18 months	Unilateral	Non-advanced	GroupD	cT2b
P31	M	14 months	Unilateral	Non-advanced	Group D	cT2a
P32	M	25 months	Unilateral	Non-advanced	Group D	cT2a
P33	F	25 months	Bilateral	Non-advanced	Group D	cT2b
C1	M	2 month		Multiple organ dysfunction (No ocular complications)		
C2	F	12 month		No ocular complications		
C3	M	3 month		No ocular complications		
C4	M	6 month		No ocular complications		
